# Development of a chemometric platform for mid-infrared fermentation monitoring

**DOI:** 10.1038/s41598-026-64638-x

**Published:** 2026-08-17

**Authors:** Mario A. Torres-Acosta, Naresh Mohan, Yiheng Yang, James Allen, John M. Ward, Gary J. Lye

**Affiliations:** 1 Tecnológico de Monterrey, School of Engineering and Science, Av. Eugenio Garza Sada2501 Sur, C.P, 64849 Monterrey, N.L México; 2 Department of Biochemical Engineering, University College London, Gower Street, London, WC1E 6BT UK; 3 Center for Biomedical Innovation, Massachusetts Institute of Technology, Cambridge, MA02142 USA; 4 Twig Bio Limited, 2 Royal College Street, Camden, London, NW1 0NH UK; 5 Manufacturing Futures Lab, University College London, Marshgate Building, 7 Sidings St, London, E20 2AE UK

**Keywords:** Chemometrics, Mid-infrared, Baseline correction, Continuous fermentation, Citramalic acid, Biological techniques, Biotechnology, Computational biology and bioinformatics, Engineering, Microbiology

## Abstract

Industrial bioprocess development still depends heavily on off-line analytics such as HPLC, which, while accurate, are slow and limit real-time decision-making. Process Analytical Technologies (PAT) can overcome this barrier, with mid-infrared spectroscopy (mIR) offering fast, sensitive, and cost-effective monitoring. Yet, most applications rely on complex chemometric tools such as Partial Least Squares (PLS), restricting adoption by non-specialists and limiting systematic evaluation of data sources. Here, we present a streamlined platform for rapid spectral analysis and chemometric model construction to monitor glucose consumption and citramalic acid production during continuous E. coli fermentations. The workflow integrates baseline correction, optimal wavenumber region selection, and correlation of solute concentrations with the area-under-the-curve (AUC), enabling straightforward model generation without advanced statistical expertise. Comparative evaluation across pure solutions, synthetic mixtures, and fermentation broths revealed continuous cultures as the most robust calibration dataset. While the baseline/AUC method showed slightly higher mean absolute error (~ 0.65 g L⁻^1^) than PLS, it provided accurate, accessible, and fast predictions consistent with HPLC values. This study demonstrates a practical and user-friendly mIR-based platform/methodology that lowers technical barriers to PAT implementation, accelerating adoption of real-time monitoring in industrial continuous bioprocesses.

**Supplementary Information:**

The online version contains supplementary material available at 10.1038/s41598-026-64638-x.

## Introduction

Bioprocesses are essential for the generation of products by microbial fermentation^1^. The fermentation process can have different components for each product generated, microbial strain used, and substrate required while their monitoring and, subsequent, control is needed^2^. Typically, monitoring is performed by taking a sample from the fermenter which is then analyzed in a separate facility (e.g., analytical laboratory). Depending on the technique performed, this can be time- and material-consuming hence the desire is to be able to perform the analysis of samples *in-situ* and in real-time^3^. This approach is known as on-line monitoring, where sample analysis is happening on the production line without removal while performing an instant analysis^4^. This results in the determination of the status of the process in real-time. This is the basis of current Process Analytical Technology (PAT) initiatives supported by the FDA (U.S. Food and Drug Administration 2004), which are designed to enable faster process development and real-time process control and optimization.

On-line and real-time monitoring can only be performed if the analytical technique employed is able to constantly generate data^4^. One of the most suitable approaches for this is spectroscopy, where a non-destructive beam of light is passed through a sample and their interaction is used to determine the composition of the sample being analyzed^5^. In this vein, infrared spectroscopy (IR) has been used to determine the chemical composition of a sample using the absorption of infrared light by chemical bonds^4^. Moreover, this property can also be used to correlate the concentration of a chemical with the amount of light absorbed. A literature review^6^ published before shows that across modelling approaches, the adopted algorithm for model creation is not consistent and its ultimate selection relies on data acquired, sensitivity of sensors, mathematical procedure, among other factors. Therefore, for new bioprocess development, it is critical to have platforms for rapid data analysis (or its evaluation), model creation, and/or subsequent concentration prediction to facilitate monitoring of fermentation in real-time new bioprocesses development and products of interest, it is critical to have a platform that is capable of data analysis, model creation, and subsequent concentration prediction to facilitate the monitoring of fermentation in real-time.

In terms of microbial fermentation, current on-line technology enables the routine monitoring of pH, liquid level, dissolved oxygen (DO), temperature and, recently, biomass (either measuring optical density or viable cells)^7^. If something else is desired to be monitored, such as a carbon source, another nutrient or product concentrations, they need to be measured elsewhere, typically through chromatography (HPLC) or specific assays^8^. For on-line monitoring of solute concentrations in microbial fermentation, near-infrared spectroscopy (nIR) has previously been used to^9^. Most recently, mid-infrared spectroscopy (mIR) probes have been developed^10^. Compared to nIR, mIR offers a number of potential advantages including less convoluted data (requiring less data processing) and higher sensitivity^3,10^.

Parallel to this, there is particular interest in generating bio-based alternatives to chemicals normally obtained from the petrochemical industry^11^. Moreover, to decrease their production cost and have a constant output, continuous fermentation is an interesting approach to constant product generation^12^. Furthermore, in order to control the cellular growth and productivity in a continuous fermentation, there is a need to have a limiting substrate (i.e., glucose-limited fermentations). In theory, the concentration of a chemical species that is present in the feeding will remain constant as the volume and concentrations fed are not changing during the steady state of the fermentation. In practice, some variation can exist due to volume changes during sample taking or an improvement on strain fitness. Moreover, from an industrial and economic perspective, having a fermentation process that wastes a high amount of nutrients becomes an expense that can be reduced by media optimization^13^. All the needed experiments to determine the best conditions in a fermenter, mainly to improve production, can be enhanced and expedited when using on-line and real-time monitoring.

For this reason, the objective of this work is to develop a data analysis and modelling platform for the rapid development of correlation models using mid-infrared (mIR) and apply it to monitor a continuous fermentation producing citramalic acid (or citramalate, CM) as a case study. This work focuses on the construction of the data analysis and modelling platform by testing the identification of ideal wavenumber regions for the prediction of the chemical species monitored (instead of using the full spectrum data), then it performs a contrast on different calibration dataset sources to determine the best alternative (single chemical solution, synthetic mixtures, and fermentation samples). Then, batch and continuous fermentation samples are employed to test the real-life applicability and implementation of the tool developed here. Lastly, the correlations were contrasted against predictions using Partial Least Squares (PLS) as a conventional technique employed in the area of chemometrics.

## Materials and methods

### Experimental materials and methods

#### Chemical and biological materials

To create the different correlation and prediction models a range of chemicals were employed to determine the extent of identification through mIR. These were glucose, glycerol, citramalate (CM), ethanol, lactate, and acetate, all acquired from Millipore-Sigma (Merck, Germany). All were used during chemometric calibration for this work or as standards for HPLC quantification, specific concentrations used for each for calibration datasets will be presented in subsequent sections. An *E. coli* strain was employed in this work that is able to produce citramalate synthase (for the production of citramalate) and a red fluorescent protein (RFP) as reporter gene, both under the same expression cassette. The development of this strain has been described before^8^^,^^14^.

#### Spectral data acquisition

The equipment employed in this work for the acquisition of mIR spectral data was the IRmadillo from Keit Spectrometers (Oxfordshire, UK). Briefly, the spectrometer has a spectral range of 800 – 4000 cm^-1^ (equivalent to a wavelength range of 2.6 – 12.5 µm), data acquisition is based on attenuated total reflection (ATR) and contains a probe that can be inserted directly into large-scale bioreactors. For laboratory applications and small vessels, the probes can be modified to attach a flow-through module. For batch fermentations, sampling occurred mainly off-line (taking a sample and injecting by pipette to the flow cells), while for continuous fermentation this was done by connecting the outlet of the fermenter to the flow-through cell of the mIR device. Fermentation protocols are explained in the next subsections. The probe contains a chalcogenide-based crystal at the tip. Before and during operation it requires a constant flow of nitrogen gas (approximately 1 L min^-1^) to remove carbon dioxide and water vapor that can interfere with mIR absorbance. For off-line spectral data acquisition (regardless of the sample being analyzed), the equipment was configured to perform 7 scans for 1 min, then perform this procedure 3 times and provide a spectral average of the 3 measurements (21 total scans per sample). This was considered as a single spectrum. For on-line spectrum acquisition, the sampling consisted of performing 7 scans for 1 min with a sample being analyzed between 2 to 5 min depending on the projected length of the experiment (resulting in a total of 14 to 35 scans per sample).

#### Fermentation protocol

For the fermentation procedures performed here, LB and minimal media (MS) were employed with the following composition. LB media contains 10 g L^-1^ of tryptone, 5 g L^-1^ of yeast extract, and 5 g L^-1^ NaCl. MS media is prepared by three individual solutions (MS-A, MS-B, and MS-C) and mixed after autoclave sterilization. MS-A contains 2 g L^-1^ of KH_2_PO_4_ and 2 mL L^-1^ of a trace element solution, MS-B contains 4 g L^-1^ for fermenters and 3 g L^-1^ for inoculum of NH_4_Cl and 0.4 g L^-1^ of MgSO_4_·7H_2_O, and MS-C contains 5 g L^-1^ of glucose. The trace element solution used for the preparation of MS-A contains: 50 g L^-1^ EDTA, 2.2 g L^-1^ ZnSO_4_, 5.54 g L^-1^ CaCl_2_, 5.06 g L^-1^ MnCl_2_·4H_2_O, 5 g L^-1^ FeSO_4_·7H_2_O, 1.1 g L^-1^ (NH_4_)_6_Mo_7_O_24_·4H_2_O, 1.57 g L^-1^ CuSO_4_·5H_2_O, and 1.61 g L^-1^ CoCl_2_·6H_2_O^15^. Media used in the vessel and feed for continuous fermentation had the same composition. Potassium phosphate buffer used in MS media at flask level was 40 mM with a pH 7. Final Kanamycin concentration for all applications used was 50 µg mL^-1^. All chemicals for media preparation were obtained from Sigma-Aldrich (MO, USA) and NH_4_Cl from Merck (Germany).

For the acquisition of fermentation samples for mIR calibration and its subsequent tests a series of batch and continuous fermentations were performed. The general protocol for fermentation and inoculum acquisition was based on previous reports^14^. The bioreactors used in this work are from Applikon and were used with the my-Control system. Vessels have a total volume of 1.2 L (working volume used of 0.9 L). The vessels contain 2 Rushton turbine impellers separated by 4.5 cm and 2 vertical baffles. General conditions for bioreactor fermentations, regardless of the mode of operation, were at 37 °C, 400 rpm and pH 7. Additionally, dissolved oxygen (DO) was controlled at 30% saturation and sterile antifoam (propylene glycol – PPG) was added only when foam was formed to avoid potential interferences with measurements. Regardless of the operation mode, the process to obtain the inoculum started with cells grown on a solid Luria–Bertani (LB) agar plate containing Kanamycin and grown overnight at 37 °C. From here, a single colony was inoculated into liquid LB containing Kanamycin and grown for approximately 12 h at 37 °C and 250 rpm,this resulted in an OD > 4. Afterwards, a sample of this LB culture was used to inoculate a flask containing MS medium with an initial OD of 0.1, then it was grown at the same conditions until the optical density achieved a reading > 0.5. The same MS medium composition was used for all bioreactor studies because it has already been proved to limit growth based on carbon restriction^14^. One drawback of the MS medium is that pH is easily affected due to acid metabolite formation, for this reason a phosphate buffer was added. After the required OD was achieved, fermenters were inoculated at an initial OD = 0.1. For batch fermentation, the process was left unmodified from this point. Meanwhile for continuous fermentation, a dilution rate of 0.1 h^-1^ (equivalent to a flowrate of 1.5 mL min^-1^) was employed and started 6 h after inoculation.

#### HPLC measurements

For quantification of glucose and CM during fermentation sampling, 1 mL was taken at each time point, centrifuged at 4,000 g for 10 min. Then the supernatant was passed through a 0.22 µm filter and analyzed by HPLC using an Aminex column (Bio-Rad Laboratories Inc., USA) coupled with a refractive index (RI) detector and operated at 60 °C. A mobile phase of 0.1% v/v of trifluoroacetic acid was used with a flowrate of 0.6 mL min^-1^. The chemical compounds to be analyzed here were glucose and citramalate with retention times expected at 9.054 min ± 0.079 min and 9.872 ± 0.082 min, respectively.

## Analysis and modelling platform development

### Platform development rationale

To construct a fast and user-friendly modelling platform a straightforward and easily understandable approach was undertaken. Given the nature of mIR absorbance it is possible to pinpoint specific ranges of wavenumbers that correspond to specific molecules (instead of single chemical bonds). For this reason, a recently reported baselining approach^16^ based on Excel (MS Office, Microsoft, Redmont VA) was adapted to work with mIR spectral data.

**Fig. 1:**
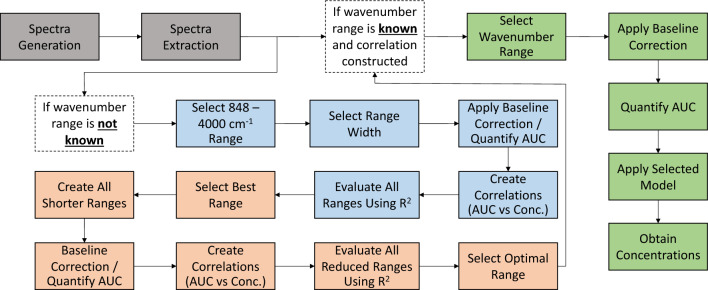
Flowchart of the data processing methodology developed in this work. Boxes in grey occur inside the IRmadillo equipment, blue boxes are for wavenumber range identification (Supplementary material 1), orange boxes are for wavenumber range optimization (Supplementary material 2), and green boxes are for the subsequent conversion of spectra into solute concentrations after model construction.

Briefly, the flow of data and its processing consists of the input of spectral data, then a specific wavenumber range is selected (procedure to identify optimal ranges to presented in subsequent sections), afterwards a baseline correction is applied to the selected region, and the area on under the curve (AUC) is quantified. Using an already computed correlation model (also explained in later sections), the AUC is converted to concentration values. A diagram to show the data processing for model construction, wavenumber range selection, and new sample processing is shown in Fig. 1. The complete analysis and modelling platform constructed for this work consists of 3 parts. The first part includes a model construction tool, the second part is used for wavenumber selection, and the third part is for sample processing (conversion of raw spectral data to chemical concentrations) and all are included in Supplementary material 1–3. Supplementary material 1 and 2 includes screenshots of the Excel platforms used for wavenumber region identification and region optimization (reduction of wavenumber range), respectively. Material supplementary 3 shows a representative spectra collection of CM for concentration between 0 and 16 g L-1, a zoom-in for a specific range, spectra after baseline correction, and correlation of AUC vs concentration.

The mathematical procedure to obtain a concentration value from a spectrum is as follows. As this method is based on a baseline adjustment, first it needs 2 data points to be selected in the spectrum which correspond to the minimum and maximum wavenumbers for the range selected along with their corresponding absorbance values. Then, a straight line (normal calculation for a slope and intercept) is calculated between these 2 points. This linear equation is used to adjust the baseline of the absorbance only for the section of wavenumbers selected and applied to each wavenumber within the range using Eq. 1.

1 $$Corrected\,Absorbanc{e}_{i}=Original\,Absorbanc{e}_{i}-(Wavenumbe{r}_{i}*Slope+Intercept)$$

After performing the baseline correction, the AUC is calculated by the sum of all the corrected absorbance values with Eq.   2.

2 $$AUC=\sum_{i=1}^{n}Corrected\,absorbanc{e}_{i}$$

where “i” represents each individual wavenumber inside the selected range.

Then the AUC calculated can be converted into concentration by applying a previously constructed correlation between AUC and different concentration of the chemical analyzed. The procedure to obtain these regressions is explained in the next section.

Wavenumber range selection and optimization.

To construct an appropriate regression model to be used to convert AUC to concentrations it is important to determine the wavenumber range ideal for each chemical analyzed. For this, calibration samples for different chemical were employed: glucose, glycerol, CM, ethanol, lactate, and acetate, all of them in a range of concentrations from 0 to 16 g L^-1^ (comprising 8 datapoints/concentrations in each case; 0, 0.5, 1, 2, 4, 6, 8, and 16 g L^-1^). Firstly, a screening was performed selecting a width of 100 wavenumbers (i.e., for wavenumbers (cm^-1^): 848 – 948, 852 – 952, 856 – 956… 3900 – 4000) to correlate AUC vs concentration evaluating the regression coefficient (R^2^). Given the nature of the IRmadillo, it has a resolution of 4 cm^-1^, with 848 cm^-1^ being the lowest wavenumber and 4000 cm^-1^ the highest.

**Fig. 2:**
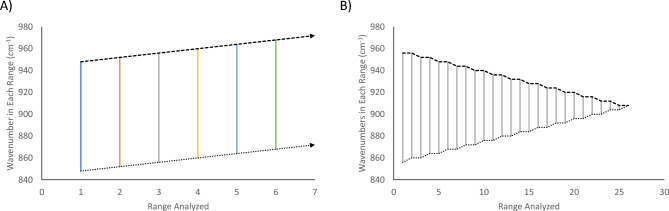
Diagrams representing the sequential analysis of different wavenumber ranges for: (**A**) wavenumber range identification, and (**B**) wavenumber range optimization. Dashed lines indicate the low and high boundaries of each range being analyzed. In (**A**) both dashed lines point towards the upper right; this means that both limits are moving in the same proportion in the same direction, which indicates the analysis of sequential ranges. In (**B**), the dashed lines converge which indicates that each range analyzed is smaller than the previous ranges. Wavenumber ranges included in (**B**) are an arbitrary example and the protocol can be utilized for any range identified in (**A**).

Once a specific range is selected accordingly to R^2^, the next step is to optimize that range by reducing its size (initial size of 100 wavenumbers). To perform this, a similar approach was undertaken, but instead of performing a panning over the whole spectrum, individual wavenumbers were removed from the extreme sides, one side at a time. For example, for a hypothetical range of 852 – 952 cm^-1^, the next step will be to analyze 856 – 952 cm^-1^, then 856 – 948 cm^-1^, then remove again from the left side to get 860 – 948 cm^-1^, then 860 – 944 cm^-1^, and so on. Both strategies for the initial selection of a range and its further optimization are presented in Fig 2. Figure 2A shows how the analyzed wavenumber window is sliding 4 cm-1 each iteration. This analysis will allow to find the one wavenumber region that has the highest R^2^, while the second approach (wavenumber window reduction) in Fig 2B will allow to see if reducing the data per window actually improves the correlation. It is important to note that window reduction is necessary to reduce computing power required, but from a correlation perspective it might not be recommend. The rationale behind this is that the less amount of data the more precise a correlation can be, but too few data points could also not capture the correct correlation between AUC and concentrations. So, this optimization of wavenumber ranges will analyze R^2^ until it drops below the initial value. At that point, the optimal wavenumber range will be selected.

### Correlation (Calibration and test) datasets

Additional to the selection of an optimal wavenumber range, this work also analyzed the impact of different calibration dataset sources. The initial dataset consists of single-chemical solutions, at this stage the dataset consisted of glucose, glycerol, CM, ethanol, lactate, and acetate, all of them in a range of concentrations from 0 to 16 g L^-1^ (comprising 8 datapoints/concentrations in each case; 0, 0.5, 1, 2, 4, 6, 8, and 16 g L^-1^). The next step was to test mixtures of chemicals. Due to the focus of this study, it was decided to analyze only mixtures of chemicals that are present in the fermentation being investigated. Thus, mixtures of glucose, CM, and biomass were examined. These solutions were prepared in a range of 0 to 16 g L^-1^ (comprising 4 datapoints/concentrations for glucose, citramalate, and biomass; 0, 4, 8, and 16 g L^-1^ for all variables.) moreover, all possible combinations were tested which resulted in 64 combinations/solutions to be analyzed (4 concentration levels and 3 possible chemicals generate 64 combinations). For single-chemical and mixture solutions, chemicals were dissolved in fermentation media (MS media) as described before without added glucose to account for potential background noise in the signal.

The last step was to test actual fermentation samples at different concentrations (depending on the time point of the process), these last samples were obtained from two sets of fermentations, one operated in batch mode and an additional one in continuous mode (batch mode data contained 4 replicates, while continuous 3 replicates). The analysis was performed having two configurations for fermentation-based samples; Option 1 was configured to have the batch fermentation as a calibration dataset, while the continuous mode as a test dataset. Inversely, Option 2 relied on continuous fermentation as calibration and batch mode as a test. Lastly, both fermentations were also analyzed in real-time by connecting a sampling loop directly into the IRmadillo and their spectra collected. The length of this loop was calculated to account for a 3 min residence time throughout the whole loop with a dilution rate of 0.1 h^-1^. After all analyses were completed, on-line data was used as the ultimate test to the models created in this work. All samples, but especially the fermentation samples, need to be quantified through HPLC to determine their concentrations and perform a better correlation.

**Fig. 3:**
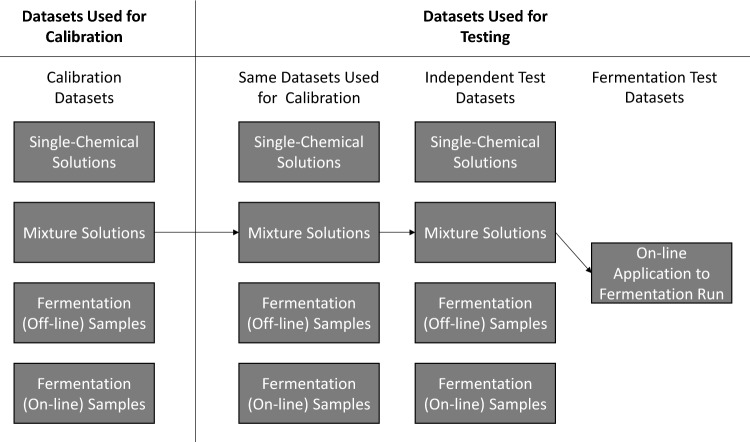
Representation of the different datasets used for model construction (calibration) and testing. Tests for each model comprised of a self-prediction to check that the model was constructed correctly, then a test with an independent set of samples prepared in the same manner as the corresponding calibration dataset, followed by a test using off-line and on-line fermentation samples in order to contrast all the models. The black arrows connecting the “Mixture Solutions” box with the others is an arbitrary example of the data flow.

After correlation models are created using the datasets described here and the procedure mentioned in the previous section, it is necessary to perform tests on the models using test datasets. For this, each type of solution used for calibrations (single-chemical, mixtures, or fermentation samples) had a parallel and independent set of samples to be used as tests. After the model/correlation creation and wavenumber range selection, all were tested to demonstrate correlation with new fermentation samples. This methodology is depicted in Fig. 3.

To compare the efficiency of the use of different calibration datasets, the Mean Absolute Error (MAE) was calculated using Eq.   3. The MAE allows to determine the average deviation that the prediction values have to the actual values determine through HPLC.

3 $$Mean\,Absolute\,Error\,(MAE)=\frac{\sum_{i=1}^{n}\left|Predicted\,Value-Actual\,concentration\right|}{Number\,of\,Samples}$$

### Partial least squares (PLS) contrast

To ultimately contrast the efficiency of the platform developed here, the results will be compared against predictions performed using PLS. To calculate PLS predictions, the open-source software R (R version 4.1.2, http://www.r-project.org) containing the “pls” package (pls version 2.9–0, https://cran.r-project.org/web/packages/pls/index.html) was used. For validation, the “leave one out” method and a “One Sigma” method for component (latent variable) number selection were employed. The number of latent variables was determined by the minimum root mean square error calculated. For PLS model construction, the same calibration and test datasets were used as for the baseline-based models. Additionally, a contrast was made between using the full spectrum for each sample or the optimized wavenumber ranged as determined by the methodology in previous sections. The approach with the lowest RMSE was selected. Two sets of predictions were constructed, the first was a cross-validation using the leave-one-out method to determine the concentrations of the datasets used for model calibration and a test prediction for validation to determine the concentrations of an independent test dataset not seen by the algorithm during model training. Contrast between both modelling approaches was performed with the MAE calculated using Eq.   3.

## Results

### Application of the analysis and modelling platform to identify optimal wavenumber ranges

The creation of a rapid and user-friendly modelling platform using mid-infrared spectroscopy (mIR) based solely on baseline correction was achieved through the development and integration of two complementary methods. The first involved the identification and optimization of useful wavenumber ranges, while the second focused on generating regression models using the AUC method previously described. Once developed independently, both components were integrated into a single platform capable of processing large spectral datasets (up to 1,000 spectra) for either individual samples or time-series data.

To determine the optimal wavenumber ranges, six chemical species were analyzed individually, and their respective optimal ranges for concentration determination are presented in Table 1. This initial screening used pure solutions to identify relevant spectral regions before moving to more complex datasets. Screenshots illustrating the wavenumber range selection and optimization procedures are included in Supplementary material 1 and 2. The ability to perform a rapid, almost instantaneous scan across the full mIR range (848–4000 cm⁻^1^) allowed efficient identification of key wavenumber regions for model construction.

**Table 1:** Wavenumber ranges selected and subsequently optimized for quantification of each of the chemical compounds studied. Wavenumber ranges were obtained by analyzing single-chemical solutions within a concentration range (0 – 16 g L^-1^) to avoid interferences from other chemicals present. Not all the wavenumber ranges required optimization and hence some values remain the same.

	Wavenumber ranges (cm^-1^)	
	Initial selection	After optimization
Glucose	932 – 1,032	932 – 1,032
	992 – 1,092	992 – 1,092
Glycerol	1,048 – 1,100	1,048 – 1,100
Citramalate	1,364 – 1,432	1,390 – 1,406
	1,532 – 1,604	1,558 – 1,578
Ethanol	976 – 1,048	988 – 1,032
	1,004 – 1,124	1,028 – 1,096
Lactate	1,068 – 1,136	1,080 – 1,124
Acetate	1,344 – 1,416	1,364 – 1,392
	1,376 – 1,456	1,404 – 1,424

**Fig. 4:**
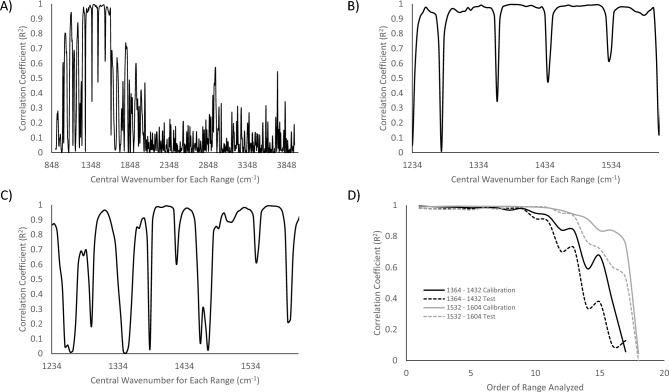
Evaluation of wavenumber ranges for citramalate model construction. (**A**) shows the initial evaluation R^2^ of different ranges with a width of 100 cm^-1^ across the whole mIR range (848 – 4,000 cm^-1^). (**B**) depicts a zoom-in of the x-axis from 1,234 to 1,606 cm^-1^. (**C**) shows an analysis of the range indicated in (**B**) but with a width for each range of 52 cm^-1^ Instead of 100 cm^-1^ as in (**B**). Finally, (**D**) shows evaluation of the optimization for both initial ranges selected (1,364 – 1,432 cm^-1^ and 1,532 – 1,604 cm^-1^). This figures also includes the R^2^ values for calibration and test datasets.

Applying baseline correction and direct AUC correlation across the studied concentration ranges allowed the calculation of straightforward linear regression models. The analysis evaluated 764 ranges per compound (100 cm⁻^1^ wide intervals, 4 cm⁻^1^ spacing with 96 cm-1 of overlap), making it possible to quickly identify regions with high correlation coefficients (R^2^). For example, in the case of citramalate (CM), Fig. 4A shows how R^2^ values vary across different wavenumber ranges, indicating where CM molecules strongly absorb infrared light. A more focused examination of the 1,234–1,606 cm⁻^1^ region (Fig. 4B) revealed that adjusting range width improves correlation definition (Fig. 4C). The final optimized regions for CM were 1,390–1,406 cm⁻^1^ and 1,558–1,578 cm⁻^1^ (Fig. 4D).

This procedure was repeated for all six chemical species (Table 1). Using single-compound solutions provided clarity in identifying distinct absorption bands for each compound. However, when using complex samples such as mixtures or fermentation broths, additional overlapping signals could require a broader analytical strategy. This strategy established a solid basis for subsequent comparison of different calibration dataset sources.

### Comparison of different calibration dataset sources

After identifying the optimal wavenumber ranges, the next step was to evaluate how the composition of calibration datasets influenced model generation and prediction accuracy. Three types of datasets were examined: single-chemical solutions, mixed chemical solutions, and actual fermentation broth samples. All spectral data were collected off-line, with samples manually introduced into the mIR cell for measurement.

The fermentation system used in this study relied on an engineered *E. coli* strain producing citramalate (CM) from pyruvate^8,14^. This strain lacks lactic acid production^8^, and acetate formation was confirmed to be negligible. Thus, glucose and CM were the only measurable solutes of interest, making them the focus of this section. Continuous fermentation was prioritized as a calibration scenario because of its relevance to industrial bioprocesses and higher data consistency. Moreover, the optimal wavenumber ranges for glucose and CM did not overlap, removing the need for complex deconvolution procedures.

**Fig. 5:**
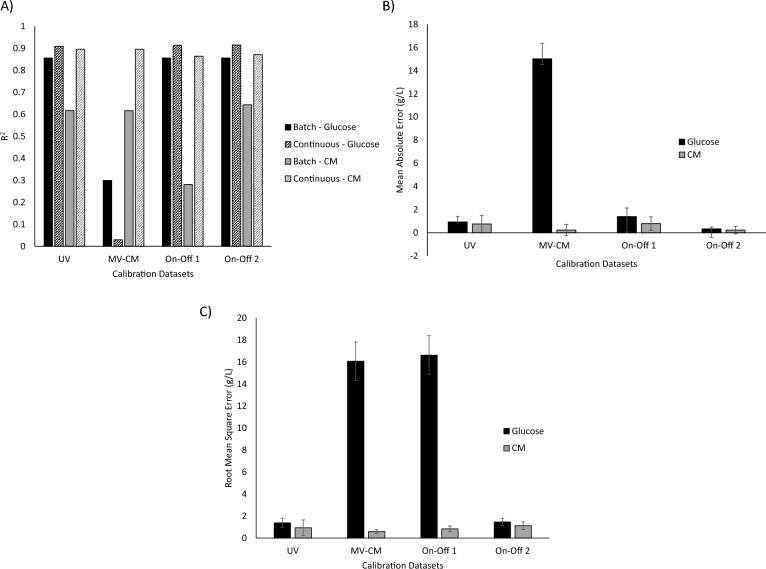
Contrast between different calibration datasets and their ability to predict solute concentrations in fermentation broth samples. (**A**) Depicts correlation coefficients (R^2^) between mIR predicted values and HPLC measurements for glucose and CM in batch and continuous fermentations. (**B**) Shows the Mean Absolute Error (based on Eq. 3) calculated for the prediction of glucose and CM in a continuous fermentation. UV: single-chemical model, MV-CM: mixture-based model, On–Off 1: fermentation-based model option 1, and On–Off 2: fermentation-based model option 2.

Regression models were generated using the previously optimized wavenumber ranges, correlating AUC values with solute concentrations across all dataset types. Each model’s performance was quantified using R^2^ values (Table 2) for predicting the same dataset used for calibration and a dataset for testing but all obtained in parallel during experimentation (R^2^ was calculated using a linear regression between predicted values vs HPLC quantification). For model selection and prevention of data leakage, each was validated against a new and independent experimental (4 batch and 3 continuous fermentations) dataset of batch and continuous fermentation with HPLC measurements was ground truth (Fig. 5). Figure 5A shows that the fermentation-based model (Option 2: continuous fermentation as calibration, batch as test) achieved the highest R^2^ values. Together, MAE values (Fig. 5B) and RMSE (Fig. 5C) confirmed that this model also had the lowest deviations and consistent variation among these indicators, indicating superior predictive accuracy when faced against new and independent samples. For this reason, this model was selected for subsequent analysis. Lastly, residuals plot for the fermentation used for validation was included in Supplementary material 4 for contrasting all calibration dataset. From this, the fermentation-based model (Option 2) presented the most consistent residuals spread across a value of zero.

**Table 2:** Model parameters and fit obtained with different calibration materials (single-chemical solutions, mixtures, and fermentation broth samples) to predict glucose and citramalate concentrations. Although the majority of them present acceptable correlation coefficients, the best option after testing against an independent fermentation was a calibration model established using fermentation broth from a continuous fermentation.

Model type	Chemical	Wavenumber range (cm^-1^)	Slope	Intercept	R^2^ calibration	R^2^ Test
Single-Chemical	Glucose	932 – 1,032	-0.00399	-0.15458	0.86	0.77
		992 – 1,092	0.004195	-0.05507		
	Citramalate	1,364 – 1,432	0.003014	-0.00292	0.96	0.92
		1,548 – 1,584	0.001701	-0.0283		
Mixtures	Glucose	932 – 1,032	-0.00344	-0.16016	0.95	0.75
		992 – 1,092	0.004525	-0.06149		
	Citramalate	1,364 – 1,432	0.002423	-0.00431	0.99	0.99
		1,548 – 1,584	0.001365	-0.02884		
Fermentation (Option 1)	Glucose	932 – 1,032	-0.00367	-0.1617	0.86	0.91
		992 – 1,092	0.00425	-0.06683		
	Citramalate	1,364 – 1,432	0.003622	-0.01043	0.28	0.86
		1,548 – 1,584	0.001362	-0.02657		
Fermentation (Option 2)	Glucose	932 – 1,032	-0.00477	-0.16348	0.34	0.80
		992 – 1,092	0.00559	-0.05927		
	CM	1,364 – 1,432	0.001672	-0.00552	0.61	0.80
		1,548 – 1,584	0.001379	-0.02828

These results demonstrate that models constructed from fermentation-derived datasets outperform those built from simpler datasets, owing to the increased spectral variability captured during real fermentations. The larger number of samples (24 per continuous fermentation) and the complex background matrix improved model robustness. Nevertheless, single-chemical and mixture-based models remain valuable for rapid preliminary calibration or spectral region identification when simplicity and speed are prioritized.

**Fig. 6:**
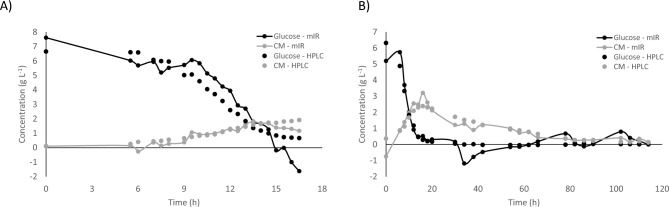
Application of the selected model (fermentation-based model – option 2) for concentration predictions during (**A**) batch and (**B**) continuous fermentations. Data shown are the average of three fermentations (error bars are not indicated for visual clarity). These graphs show that the mIR model selected is able to track the general trends of glucose depletion and CM accumulation and later production stoppage (in the case of continuous fermentation). Negative values in the Y-axis are included as some predicted values at very low concentrations can sometimes become less than zero.

The continuous fermentation performed here was not able to sustain a steady state for CM and biomass, but it did for glucose (less than 5% variation on concentration during the majority of the continuous stage), as shown in Fig. 6. This non-steady state has been analyzed elsewhere^14^. Briefly, it is hypothesized that this is caused by two phenomena: 1) concatenating of plasmid and causing an imbalance in plasmid splitting during cellular division and 2) pyruvate competition for cellular growth and CM production under a glucose-limited media. Altogether leading to cessation of citramalate synthesis. Such findings emphasize the importance of real-time or rapid monitoring methods to identify performance declines during production.

### In-Situ fermentation monitoring

After developing and validating the platform, the optimized wavenumber ranges and the selected calibration dataset (Fermentation-based Option 2) were applied to monitor both batch and continuous fermentations. Figure 6 shows glucose and citramalate concentration profiles obtained through HPLC and mIR analyses. For batch fermentations (Fig. 6A), the mIR-based predictions effectively followed the expected glucose depletion and CM accumulation trends, with MAE values of 0.89 and 0.24 g L⁻^1^, respectively. In continuous fermentation, the mIR method closely tracked glucose depletion (MAE = 0.31 g L⁻^1^) and CM present in the outlet (MAE = 0.20 g L⁻^1^), demonstrating strong consistency with HPLC data.

This ability to detect the cessation of CM production in real time is particularly significant for process optimization. Identifying the onset of productivity loss allows operators to terminate unproductive fermentations early, conserving resources and improving process efficiency. The mIR platform’s simplicity also allows near real-time interpretation without reliance on advanced analytical instrumentation.

Finally, the baseline/AUC model (Fermentation-based Option 2) was compared against Partial Least Squares (PLS) regression, the conventional chemometric standard. First, PLS utilized the full spectrum for calibration and prediction as it showed a lower RMSE compared to using the optimized wavenumber ranges used for the baseline/AUC method. This is due to the capabilities of PLS to capture interaction across multiple values and variables which make it a robust modelling tool. For each chemical analyzed here (glucose and citramalate), the number of latent variables were 9 and 7, respectively, as they obtained the lowest RMSE when analyzed through the leave-one-out method.

**Fig. 7:**
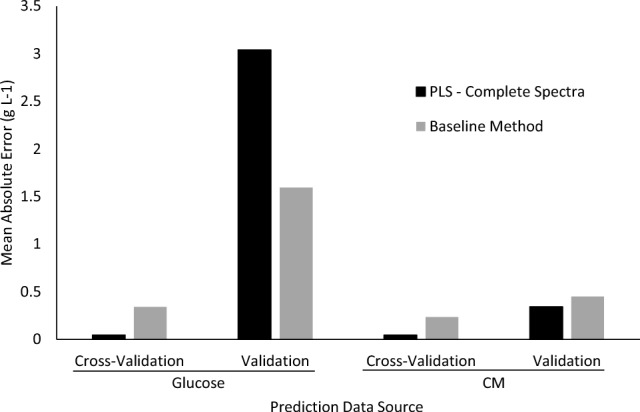
Contrast of the errors (Eq. 3) between Partial Least Square (PLS) and the baseline-based method proposed in this work for different fermentation datasets. The calibration dataset represents a continuous fermentation and the test dataset a batch fermentation. ‘Self-prediction’ indicates use the developed model to predict the dataset used for its initial construction while ‘test prediction’ is the prediction of an independent set of data not used for initial model construction.

**Fig. 8:**
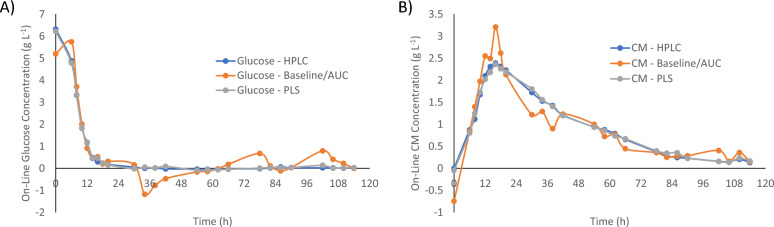
Comparison of fermentation data obtained by off-line HPLC analysis and off-line *in-situ* mIR analysis (mIR values predicted using either a baseline-based model or a PLS-based model). This data is taken from a continuous fermentation tracked over 114 h. The same samples were used to obtain HPLC results and mIR data. mIR data was subsequently employed in the construction of a PLS-based model and a baseline-AUC-based model.

Figures 7 and 8 summarize results of contrast against PLS. Figure 7 shows the contrast of predicting concentration for glucose and CM for the samples used during training and for validation (independent dataset). Results indicate lower MAE for PLS with the exception for glucose’s validation. When applying these models to track a glucose (Fig. 8A) and CM (Fig. 8B) in a continuous fermentation and contrasted against HPLC, it can be seen that PLS provide the better performance. However, the baseline/AUC approach maintained satisfactory accuracy while offering major advantages in ease of use, transparency, and speed. The platform’s implementation in Excel further enhances accessibility for non-specialists, enabling practical deployment in industrial and research settings.

Being an Excel-based algorithm, the baseline/AUC approach can be implemented by the majority of users. This also allows for all calculations to be seen as they are performed and keep record of intermediate steps, something that is not typically available through coding platforms (i.e., R or Phyton). Lastly, the AUC/baseline method requires less amount of data through the use of reduced wavenumber ranges, in contrast with PLS as it was shown here that needed the full spectrum of each sample to provide low RMSE. As more and more samples are accumulated, the use of reduce dataset lowers the computational demand.

## Discussion

The results confirm that the baseline/AUC-based mIR platform provides an efficient, user-friendly alternative for chemometric model development and fermentation monitoring. Its main strength lies in combining simplicity and speed with reliable quantitative performance. While PLS achieved slightly lower MAE values, the baseline/AUC method’s transparency, interpretability, and reduced computational burden make it well-suited for industrial users seeking rapid and accessible analytical tools.

The workflow for wavenumber optimization proved highly effective for identifying chemically meaningful regions, enabling model construction based on the most informative spectral segments. By scanning and optimizing hundreds of spectral intervals, the platform ensured the selection of robust, interpretable models without requiring multivariate complexity. The resulting wavenumber windows for glucose and CM provided accurate concentration predictions with minimal preprocessing, representing a significant step toward simplified real-time chemometric modelling.

The comparison among calibration dataset sources highlighted that models based on fermentation-derived samples were the most accurate and robust. These datasets inherently capture the complexity of real bioprocess environments, including background signals from biomass and metabolites. While single-chemical and synthetic mixtures are useful for initial screening, fermentation-based calibration ensures better model transferability to real-world applications.

The observed instability of citramalate production during continuous operation illustrates the biological and genetic limitations of engineered strains under nutrient limitation. As reported by^14^, plasmid concatenation and promoter mutations can disrupt product synthesis even when selective pressure is maintained. Such biological variability underscores the practical value of real-time mIR monitoring, allowing operators to identify process failure before productivity drops to zero.

From a process control perspective, the baseline/AUC method represents a step forward in democratizing chemometric model use in bioprocessing. The platform’s Excel-based design facilitates rapid implementation without specialized coding or statistical training, making it accessible for routine process analytical technology (PAT) applications. By minimizing data-processing complexity while maintaining high predictive reliability, it provides a valuable bridge between research-level chemometrics and industrial monitoring needs.

## Conclusions

The present work demonstrated the potential of mIR as a PAT tool for future on-line fermentation monitoring. Compared to the more well-established nIR technologies, the new range of mIR devices are able to use a simpler processing technique and can pinpoint specific wavenumber ranges per chemical specie. Building on this, the current work also showed that it is possible to develop a rational approach for the creation of chemometric models. The study consisted of the development of a platform for the construction of prediction models using a simple data processing tool. Additionally, this work contrasted the impact of using different sources of data to construct regression models. Current literature focuses on applying a specific method or source of data and there is a lack of a contrast of these. This makes the present work relevant as a platform for decision-making when designing future chemometrics modelling and on-line monitoring of fermentations.

At-line and on-line monitoring is especially relevant during continuous fermentations as nutrients are constantly fed into the vessel and the carbon-source in particular is often a major contributor to the overall cost-of-goods for chemicals manufactured via microbial fermentation. The methodology presented here was able to properly track the consumption of glucose in a substrate-limited fermentation. Moreover, as citramalate generation relies on bulk production, minimizing wastes and resources can determine if the process is viable or not. Moreover, cost can be further minimized by developing less complex and faster chemometric models as such the use of the Baseline/AUC approach developed here. This novel platform, and the detailed contrast performed here, will allow for rapid implementation of chemometrics and become a user-friendly alternative that requires minimum training. Future developments should try to incorporate actively on-line monitoring and, potentially, control platforms for all production processes to reduce costs and maximize product generation.

## Supplementary Information

Supplementary Information 1.

Supplementary Information 2.

Supplementary Information 3.

Supplementary Information 4.

## Data Availability

The datasets used and/or analysed during the current study available from the corresponding author on reasonable request.
